# Screening of radiotracer for diagnosis of colorectal cancer liver metastasis based on MACC1-SPON2

**DOI:** 10.1007/s00261-021-03015-w

**Published:** 2021-03-13

**Authors:** Hao Jiang, Wei Guo, Kuan Huang, Huijie Jiang, Rongjun Zhang, Hongbo Hu, Xue Lin, Song Wang

**Affiliations:** 1 Department of Radiology, The Second Affiliated Hospital of Harbin Medical University, Harbin, China; 2 Department of Ultrasound, Harbin the First Hospital, Harbin, China; 3 Department of Radiology, Ningbo Yinzhou No.2 Hospital, Ningbo, China; 4 Jiangsu Institute of Nuclear Medicine, Wuxi, China; 5 Department of Radiology, Longhua Hospital Shanghai University of Traditional Chinese Medicine, Shanghai, China

**Keywords:** ^18^F-FLT, ^18^F-FMISO, ^18^F-FDG, PET, Colorectal cancer liver metastasis, Biological characteristics

## Abstract

**Background:**

Metastasis-associated in colon cancer 1 (MACC1) and Spondin2 (SPON2) are newly discovered oncogenes, but little is known about their role in colorectal cancer(CRC) liver metastases. PET has become an important molecular imaging technology due to its high sensitivity and quantifiability. In particular, its targeted, specific molecular probes can detect biological behaviors. This study was designed to evaluate the different biological properties of ^18^F-FDG, ^18^F-FLT, and ^18^F-FMISO PET. The value of the CRC liver metastasis model explores the correlation and potential mechanisms of three tracers uptakes with tumor-related biological characteristics.

**Methods:**

Human CRC cell lines(LoVo and HCT8), were cultured for in vitro radionuclide uptake experiments to compare the molecular imaging features of colorectal cancer cells with different metastatic potentials. Two kinds of cells were injected into the spleen of nude mice to establish a liver metastasis model. After the tumor formation, three kinds of tracer PET images were performed to evaluate the characteristics of live PET imaging of high and low liver metastasis colorectal cancer models. The expression levels of MACC1 and SPON2 in tissues were detected by immunohistochemistry and Western blot. Correlation between tracer uptake and expression of MACC1 and SPON2 in liver metastases was assessed by linear regression analysis.

**Results:**

The uptake rate of in vitro three tracers uptake experiments was LoVo > HCT8. Micro-PET scan showed no significant difference between the ^18^F-FDG SUV values of the two cells (*P* > 0.05); there was significant difference between the ^18^F-FLT and ^18^F-FMISO SUV values (*P* < 0.05). All in vivo FLT and FMISO SUV values were significantly higher in LoVo tumors than in HCT8 tumors. The results of Western blot and immunohistochemistry showed that the expression levels of MACC1 and SPON2 in LoVo liver metastasis were higher than those in HCT8 (*P* < 0.05). The ^18^F-FLT SUVmax ratio was significantly correlated with the expression of MACC1 and SPON2 in hepatic metastases (r = 0.737, *P* = 0.0026; r = 0.842, *P* = 0.0002). The ^18^F-FMISO SUVmax ratio was only significantly correlated with the expression of MACC1 in hepatic metastasis (r = 0.770, *P* = 0.0013).

**Conclusions:**

Early screening with ^18^F-FLT and ^18^F-FMISO tracers has important clinical value for the efficient diagnosis and treatment of colorectal cancer liver metastases.

## Introduction

According to global cancer statistics in 2018, colorectal cancer (CRC) has become the fourth leading cause of death from cancer in the world, accounting for 10% of the total number of cancers diagnosed and cancer-related deaths each year, and is the second most common cancer among women and men It is the third most common cancer, causing nearly 900,000 deaths each year. The annual number of new incidences of colorectal cancer worldwide is expected to increase to 2.5 million in 2035 [1, 2]. The main cause of death of CRC is liver metastasis. From an anatomical point of view, the draining venous blood of the colorectal all converges into the portal vein and preferentially flows into the liver, the liver sinusoid is the part of the gastrointestinal tract where blood returns. The liver has a high clearance rate for blood flow, so it is the organ where tumor cells are most likely to stay; the incidence of colorectal cancer invading veins is as high as 20% to 30%. Once tumor cells fall off the primary tumor and enter the blood circulation, they are easily Metastases formed in the liver. If effective treatment is not available, the probability of liver metastasis will eventually be at least 50% [3]. Therefore, to explore the genes that lead to the development of CRC, and to understand the clinical significance of these genes is crucial for the diagnosis and treatment of CRC. These key molecular characterizations are expected to help the development of new CRC therapeutic strategies. PET can display biological features within tumors in vivo at functional and molecular levels by means of targeted probes. We can use this characteristic of PET to evaluate the biological characteristics (invasion and metastasis) of tumors, and to identify the high invasion and metastasis characteristics of colorectal cancer in advance, which is helpful for the appropriate treatment of liver metastasis of colorectal cancer.

It is well known that the microenvironment of tumors leads to numerous physiological consequences, including invasion and metastasis. ^18^F-FDG is currently the most widely used PET targeting probe. ^18^F-FDG PET has become an indispensable means of examination for many types of tumors, including CRC. However, numerous studies have shown that ^18^F-FDG reveals that the ability of many tumor and non-tumor biological processes is not completely reliable [4, 5]. At the same time, hypoxia can lead to an increase in tumor progression by promoting glycolysis, angiogenic factor activity, metastasis, mutation, and by inhibiting apoptosis [6]. The location and extent of hypoxia in solid tumors of different types/different stages are essential for clinical treatment of cancer. ^18^F-FMISO is a common tracer for hypoxic imaging. In addition, the occurrence of malignant tumors is often due to rapid proliferation of cancer cells and formation of new blood vessels. 3′-deoxy-3′-18F-fluorothymidine (FLT) as a proliferation tracer [7], after the single enzyme thymidine kinase-1 (TK1) monophosphorylation in the DNA synthesis salvage pathway, the tracer is captured in the cytosol [8]. FLT has been found to non-invasively assess the proliferation rate of several types of tumors such as colorectal cancer, esophageal cancer, and lung tumors [8–11]. Therefore, the above three radiotracers were synthesized for PET imaging of tumors.

MACC1 is a differentially expressed gene discovered by Stein et al. [12] in 2009 through a genome-wide search for primary and metastatic colon cancer, which promotes metastasis of colon cancer, hence the name. MACC1 is a biomarker for a variety of solid tumors including colorectal cancer tumor progression, metastasis, and patient survival [13]. It has been found that MACC1 expression in tumor lesions of CRC patients is significantly increased relative to their non-tumor adjacent tissues. High expression of MACC1 is significantly associated with tumor metastasis and poor clinical outcome, and is an early risk factor for cancer patients [14, 15]. Studies have shown that knockout MACC1 significantly inhibits proliferation, migration, invasion and tumorigenesis of colorectal cancer cells, and induces apoptosis [16]. Schmid et al. [17] showed for the first time that SPON2 is one of the main downstream effectors of MACC1, providing a new link between MACC1 and SPON2. Some scholars have identified through genome-wide expression analysis that SPON2, as one of the major downstream effectors of MACC1 gene, can be used as an independent prognostic indicator for CRC metastasis and metastasis-free survival (MSF) [12]. In addition, as early as 2013, it has been reported that SPON2 is highly expressed in prostate cancer cells, and serum SPON2 index has high sensitivity and specificity, which is suitable for tumor diagnosis [18]. In this paper, MACC1 and SPON2 were introduced to explore its mechanism of action in colorectal cancer metastasis. The correlation between multi-probe molecular imaging features of early liver metastases and MACC1 and SPON2 was determined by in vitro and in vivo experiments.

Based on the previous research [19], this study added new molecular probes (^18^F-FLT) and biological indicators (MACC1, SPON2), which are intended to conduct in-depth research on the mechanism of liver metastasis of colorectal cancer, and select new organisms. The relationship between academic indicators and various molecular imaging features will further explore the relationship between the molecular mechanism of liver metastasis of colorectal cancer and tumor biology, and provide assistance for the early diagnosis, treatment and efficacy evaluation of CRC liver metastasis.

## Materials and methods

### Cell culture

The human colorectal cancer high metastatic potential cell line LoVo and the low metastatic potential cell line HCT8 were purchased from the Shanghai Cell Bank of the Chinese Academy of Sciences. The LoVo cell line was cultured in F-12 k (Buddhist) medium, and the HCT8 cell line was cultured in 1640 (Buddhist) medium. All the medium was added with 1% cyan-streptomycin and 10% fetal bovine serum (Biological). Industries), cultured in a 5% CO_2_, 37 °C incubator.

### A mouse model of hepatic metastases

Five-week-old BALA/C female nude mice were purchased from Changzhou Cavans Animal Experiment Company. The 40 nude mice purchased were housed in a sterile animal room with a temperature and humidity of 24 °C and 55 ± 10%, respectively, and the feeding conditions were carried out in accordance with the animal house feeding regulations. Animal research was approved by the Animal Management and Use Committee (IACUC) of the XXX of Atomic Medicine. Forty nude mice were divided into two groups, LoVo and HCT8, with 20 rats in each group. The nude mice were placed in the animal room for 2–3 days, and then modeled, and the spleen was inoculated to protect the spleen. The concentration of both cells was 5.0 × 10^6^/0.15 mL, and the whole operation was performed under anesthesia (2% isoflurane). There were 18 model rats remaining in the LoVo group due to surgical trauma. After the surgery, continue to be kept in the animal room. After micro-PET imaging, the model rats were sacrificed and specimens were taken.

### Preparation of ^18^F-FDG, ^18^F-FLT and ^18^F-FMISO

^18^F-FDG, ^18^F-FLT and ^18^F-FMISO were synthesized with individual PET kits (XXX Industrial Technology and Trade Corporation, XXX) on a fluorine multifunctional synthesis module [PET MF-2 V, PET (Beijing) Technology Co. Ltd., PRC] with a computer interface. The radiochemical purity of the ^18^F-FDG, ^18^F-FLT and ^18^F-FMISO tracers was greater than 95% and the final specific activity was greater than 0.5 TBq/mmol. These ^18^F-labeled tracers were diluted with 0.9% saline and passed through a 0.22 μm Millipore filter (Millipore, Billerica, MA, USA) for administration to animals.

### In vitro cellular uptake of radiotracers

Prior to ^18^F-FDG cell uptake, LoVo and HCT8 cells were cultured in serum-free medium and placed at 37 °C, 5% CO_2_. Prior to the ^18^F-FMISO cell uptake assay, LoVo and HCT8 cells were pre-incubated at 37 °C in a 2% O_2_ hypoxic environment. Cells were not treated specially before ^18^F-FLT ingestion. When the cells were grown to log phase, the cells were counted under the microscope. In the cell uptake experiment, LoVo or HCT8 cells, radiotracer and buffer were added to a glass test tube (2 ml) and incubated in a 37 °C water bath for 30, 60, 120, and 240 min, respectively, in which the O group: 100 μl was added. Radionuclide and 200 μl buffer (containing 0.2% BSA) as control group; T group: only 100 μl of radionuclide was added for measurement of radionuclide dose; X group: 100 μl of radionuclide, 100 μl of cell suspension and 100 μl of buffer DMEM containing 0.2% bovine serum albumin. The number of cells in the cell uptake experiment was unified to 5 × 10^5^ cells/100 μl, and each experiment was repeated three times at the same time point. After incubation at different times, centrifugation (1000 rp/min, 5 min), aspirate all the liquid in the O and X group. The radioactivity in each tube was accurately measured using an automatic gamma counter (PerkinElmer, 2480, USA). The cell uptake rate was calculated as: X(cpm)—O(cpm) / T(cpm)%.

### Micro-PET imaging

Eighteen weeks after the model rats were established, ^18^F-FDG, ^18^F-FLT, and ^18^F-FMISO PET imaging were performed. Micro-PET imaging was performed in the order of ^18^F-FDG, ^18^F-FLT, and ^18^F-FMISO for a specific imaging time point, with an interval of 24 h, in consideration of ^18^F attenuation. ^18^F-FDG, ^18^F-FLT, and ^18^F-FMISO were injected at 60 min, 120 min, and 240 min before scanning, respectively. The tail vein was injected with ^18^F-FDG (about 3.7 MBq, 100 μCi), ^18^F-FLT (about 7.4 MBq, 200 μCi), and ^18^F-FMISO (about 14.8 MBq, 400 μCi). Before ^18^F-FDG PET imaging, the model rats were starved for 12 h and fasted without water. The body temperature of the mice was maintained by a heat lamp throughout the process. All small animal PET imaging was performed under the protocol approved by the Animal Management and Use Committee of the Jiangsu Institute of Atomic Medicine. 10 min before the scan, anesthetized with isoflurane. Micro-PET images were acquired using a 3D Inveon Micro-PET scanner (Siemens). Image reconstruction based on ordered subset expectation maximization using Inveon Acquisition Workplace workstation (version 2.0, Siemens), matrix: 128 × 128 × 159; pixel size: 0.86 × 0.86 × 0.8 mm; truncation frequency 1.5, uniform resolution. For image analysis, ASIProVM 6.8.6.9 image processing software (Concorde Microsystems, LLC) was used to manually outline the region of interest (ROI) to cover the entire liver metastases on the image. The maximum normalized uptake ratio is defined as the tumor SUVmax divided by the liver SUVmax. The formula for SUVmax is:

$${\text{SUVmax}} = \frac{{{\text{Max}} \times 8000{{\mu Ci}}/{{ml}} \times {{weight}}\left( {\text{g}} \right)}}{{\text{injected dose}}\,({{\mu Ci}})}$$

### Immunohistochemical staining

Liver metastasis tissue specimens were fixed in 10% formalin for 48 h, embedded in paraffin, and sliced to a thickness of 3 μm. After dewaxing, the tissue sections were boiled in 10 mmol/L citrate buffer (pH6.0) for 10 min and then cooled at room temperature for 40 min for antigen retrieval. The endogenous enzyme was inactivated with 3% hydrogen peroxide, and the sections were incubated with the primary antibody overnight at 4 °C. The primary antibody was as follows: MACC1 (Abeam, ab106579, UK) primary antibody working concentration was 1:200, SPON2 (Abeam, ab215451, UK) primary antibody working fluid concentration was 1:20. On the next day, tumor sections were then incubated with HRP-labeled secondary antibody (Zhongshan Golden Bridge Biotechnology, Beijing, China) for 20 min at room temperature followed by counterstaining with hematoxylin. Slides were then rinsed with tap water and sealed with neutral resin. The staining was observed under a BX53 Olympus microscope (Olympus) at magnification 200 × . A brown-yellow staining was defined as positive. Staining intensity was defined as follows: –, no staining; + , stained areas < 25% or weak staining; +  + , stained area within 25–75% or moderate staining; +  +  + , stained area > 75% or strong staining. The slides were examined by two independent researchers in a double-blinded manner.

### Western blot analysis

Western blot analysis can quantitatively analyze the protein expression of colorectal cancer liver metastases with different metastatic potentials. The operation method refers to the previous literature [20]. The samples were placed in 4 °C RIPA buffer for 30 min. The protein concentration of the samples was determined using a Protein Concentration Assay (BCA) kit (Beyotime Biotech). An equal amount of sample protein was dissolved in a 10% or 12% SDS-PAGE gel and blotted onto a PVDF membrane (Millipore, Billerica, MA, USA). The cells were then sealed with TBS buffer containing 10% skim milk powder (containing 0.1% Tween-20) for 1 h, followed by incubation with different primary antibodies overnight at 4 °C. The primary antibody used was the same as the immunocytochemistry experiment described above, MACC1, SPON2 (1:1000 dilution), GAPDH (1:5000 dilution, Zhongshan Jinqiao Biotechnology Co., Ltd.), and then with the peroxidase-labeled secondary antibody (Diluted 1:5000) Incubate for 1 h at room temperature. Band images were acquired using a Bio-Rad chemiDoc XRS + imaging system, normalized to GAPDH expression levels, and four protein expression levels were quantified by Image-J (NIH, Bethesda, MD, USA) software.

### Statistical analysis

All data were expressed as the mean ± standard deviation. Statistical analysis was performed using SPSS software version 19.0 (SPSS, Chicago, IL, USA). The difference between two cell lines was assessed using Student’s unpaired *t*-test. The correlation between Western blotting results and PET SUV was analyzed using linear regression. *P* < 0.05 was considered statistically significant.

## Results

### In vitro radiotracer uptake analysis

The in vitro cellular uptake differences of the ^18^F-FDG, ^18^F-FLT and ^18^F-FMISO tracers were compared between LoVo and HCT8 cells (Tables 1, 2, 3). According to the results of repeated measurement analysis, the cell uptake rates of both cells of ^18^F-FLT and ^18^F-FMISO increased with time. The difference in uptake between LoVo and HCT8 cells was analyzed by independent sample t-test. The results showed that the uptake rate of three tracers was lower in HCT8 cells than in LoVo cells at 240 min. The difference was statistically significant (*P* values were 0.001, 0.000, 0.020). Moreover, the uptake rates of the two cells to ^18^F-FDG were significantly higher than those of ^18^F-FMISO and ^18^F-FLT.

**Table 1:** Comparison of ^18^F-FDG cell uptake by LoVo and HCT8 cells in vitro (%)

	LoVo	HCT8	*P*值
30 min	11.56 ± 0.09	13.57 ± 0.82	0.013
60 min	17.42 ± 1.05	19.59 ± 3.64	0.377
120 min	25.36 ± 0.33	26.30 ± 0.73	0.114
240 min	33.77 ± 1.86	23.81 ± 0.96	0.001

**Table 2:** Comparison of ^18^F-FLT cell uptake by LoVo and HCT8 cells in vitro (%)

	LoVo	HCT8	*P*值
30 min	6.34 ± 0.20	2.13 ± 0.31	0.000
60 min	9.52 ± 0.06	3.66 ± 0.24	0.000
120 min	13.35 ± 0.47	4.85 ± 0.05	0.000
240 min	21.90 ± 0.76	12.14 ± 0.77	0.000

**Table 3:** Comparison of ^18^F-FMISO cell uptake by LoVo and HCT8 cells in vitro (%)

	LoVo	HCT8	*P*值
30 min	1.31 ± 0.32	1.22 ± 0.11	0.672
60 min	1.36 ± 0.06	1.24 ± 0.09	0.093
120 min	1.74 ± 0.12	1.55 ± 0.34	0.398
240 min	2.91 ± 0.19	2.11 ± 0.32	0.020

Tables 1, 2, 3: Comparison of in vitro ^18^F-FDG, ^18^F-FLT, and ^18^F-FMISO uptake rates for different metastatic potential colorectal cancer cell lines over four time periods. Values are expressed as ^18^F-FDG (Table 1), ^18^F-FLT (Table 2), and ^18^F-FMISO (Table 3) uptake dose percentages (% ID) per 5 × 10^5^ cells. Each cell was repeated three times at the same time point. Table 1,2,3: The cell uptake rates of the ^18^F-FLT and ^18^F-FMISO of the two cells increased with time. The uptake rate of the three tracers by HCT8 cells was only lower than that of LoVo cells at 240 min. The uptake rates of the two cells to ^18^F-FDG were significantly higher than those of ^18^F-FMISO and ^18^F-FLT.

### Micro-PET imaging analysis

**Fig. 1:**
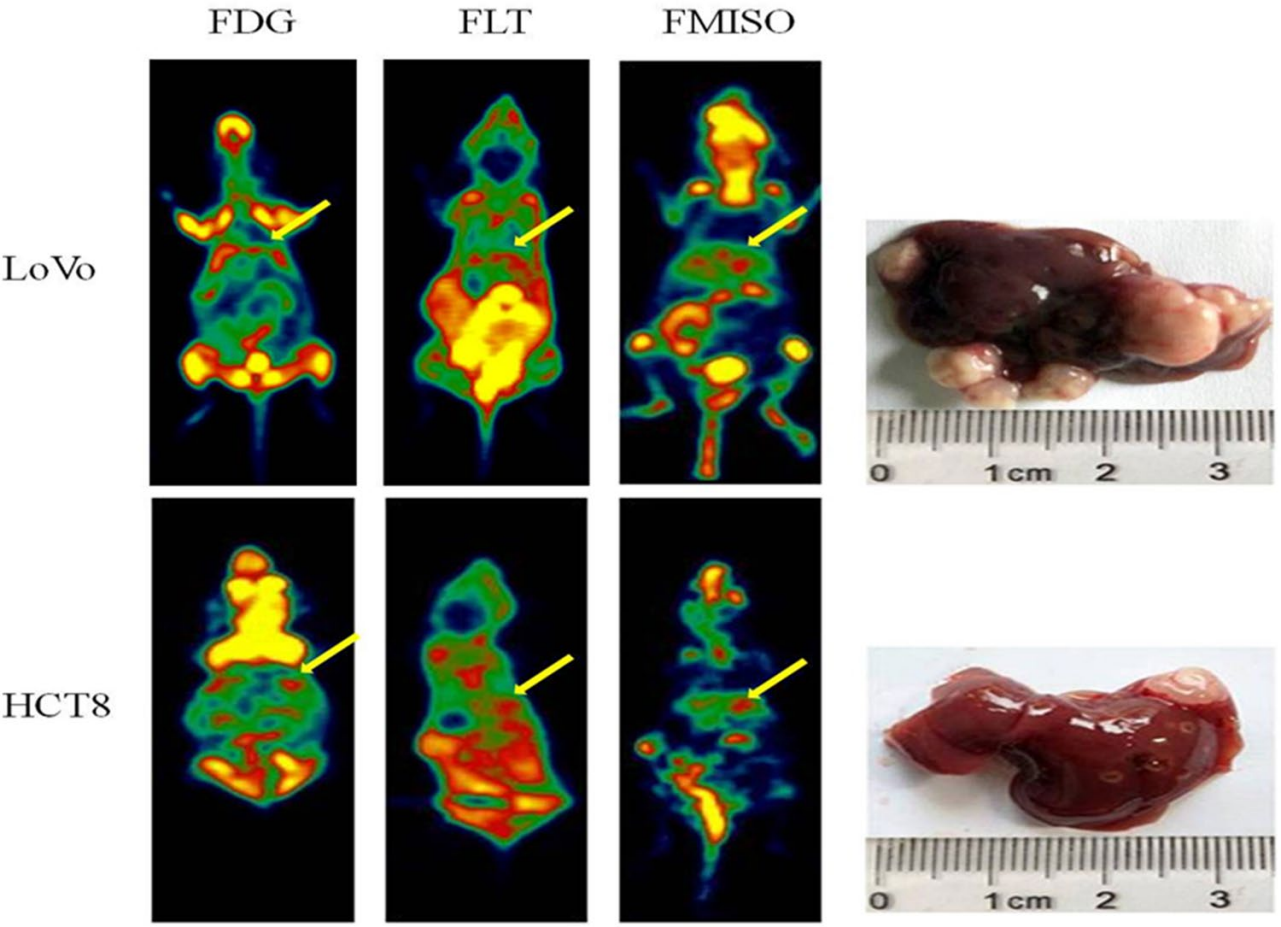
^18^F-FDG, ^18^F-FLT, and ^18^F-FMISO PET images of LoVo and HCT8 liver metastasis models and their corresponding liver anatomical images after 8 weeks of spleen injection of tumor cells. Yellow arrows indicate uptake of ^18^F-FDG, ^18^F-FLT, and ^18^F-FMISO in LoVo and HCT8 liver metastasis lesions; white nodules indicate liver metastases. It can be seen that the edge of liver metastasis lesions in ^18^F-FMISO Micro-PET imaging is clear, and the boundary between the liver and the surrounding organs is clear, and the degree of recognition is high. The edge of liver metastasis lesions in ^18^F-FLT Micro-PET imaging is blurred, intestinal tube, bladder, etc. The organ intake was higher and the recognition was relatively low. In the ^18^F-FDG Micro-PET imaging, the liver and metastases were relatively insignificant due to the high intake of the mouse head and heart

**Fig. 2:**
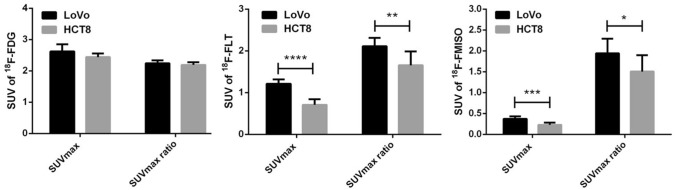
Between three tracer (^18^F-FDG, ^18^F-FLT, ^18^F-FMISO) PET parameter values (SUVmax, SUVmax ratio) in the LoVo (n = 9) and HCT8 (n = 7) liver metastasis models Compare, ****P < 0.0001

The most important issue in the establishment of a liver metastasis xenograft model is to determine the optimal time for PET imaging. The survival time of the liver metastasis mouse model is generally shorter than that of the traditional subcutaneous tumor model. Considering the time of imaging of multiple tracers, we determined the optimal imaging time as 8 weeks after tumor cell inoculation. Figure 1 shows LoVo and HCT8 models ^18^F-FDG, ^18^F-FLT, and ^18^F-FMISO PET images and their corresponding liver anatomy. The LoVo model rat liver metastasis rate is 50% (9/18), HCT8 model rat liver The metastatic rate was 35% (7/20). The difference between the three tracer SUVmax values between LoVo and HCT8 liver metastases was analyzed using an independent sample t-test. The results showed that there was no significant difference between the ^18^F-FDG SUVmax value and the SUVmax ratio of LoVo and HCT8 liver metastasis (*P* = 0.057 and 0.286) (Table 4, Fig. 2). However, there was a significant difference between the ^18^F-FMISO and ^18^F-FLT SUVmax and SUVmax ratio values of LoVo and HCT8 liver metastasis tissues, which was statistically significant (*P* < 0.05). And the ^18^F-FDG SUVmax values of the two cells were significantly higher than the SUVmax values of ^18^F-FLT and ^18^F-FMISO, which was consistent with the results of in vitro cell uptake experiments. It can be seen that ^18^F-FDG is more sensitive to liver metastases, but its specificity is poor.

**Table 4:** Comparison of FDG、FLT, and FMISO indices between LoVo and HCT8 metastatic tumors

Index	LoVo(n = 9)	HCT8(n = 7)	*P* value
FDG PET			
FDG SUVmax	2.62 ± 0.07	2.45 ± 0.04	0.057
FDG SUVmax ratio*	2.24 ± 0.03	2.20 ± 0.03	0.286
FLT PET			
FLT SUVmax	1.20 ± 0.04	0.70 ± 0.04	0.000
FLT SUVmax ratio*	2.11 ± 0.07	1.62 ± 0.11	0.002
FMISO PET			
FMISO SUVmax	0.39 ± 0.02	0.23 ± 0.02	0.000
FMISO SUVmax ratio*	1.98 ± 0.12	1.49 ± 0.13	0.014

*Metastatic tumor SUVmax divided by the liver SUVmax

### Immunohistochemical results analysis

**Fig. 3:**
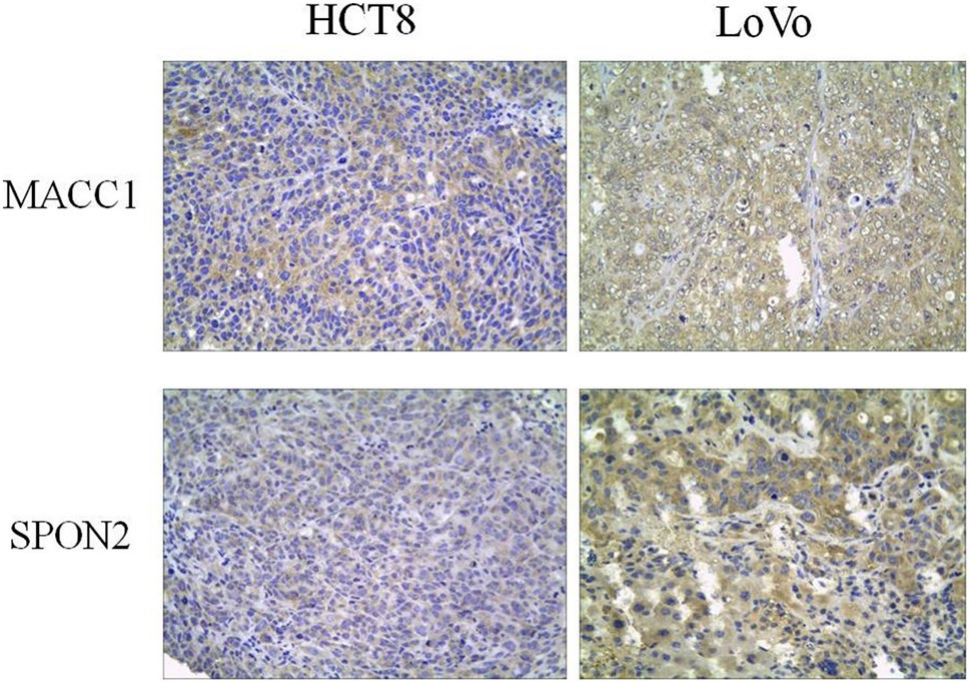
Immunohistochemical staining of human colorectal cancer LoVo and HCT8 liver metastases reflecting the level of protein expression as a result of yellow staining. The results showed that MACC1 was mainly expressed in the cytoplasm of two kinds of cellular liver metastasis tissues; SPON2 was expressed in both cytoplasm and cell membrane. The expressions of MACC1 and SPON2 in LoVo liver metastasis were significantly higher than those in HCT8 (× 400)

Figure 3 shows the expression of both MACC1 and SPON2 proteins in LoVo and HCT8 liver metastases. Immunohistochemical results showed that two models (LoVo and HCT8) had two proteins in liver metastasis, and the level of yellow staining reflected the level of protein expression. MACC1 is mainly expressed in the cytoplasm of two cell liver metastasis tissues; SPON2 is expressed in both its cytoplasm and cell membrane. The expression of both proteins in LoVo liver metastasis was higher than that in HCT8 liver metastasis (× 400).

### Western blot results analysis

**Fig. 4:**
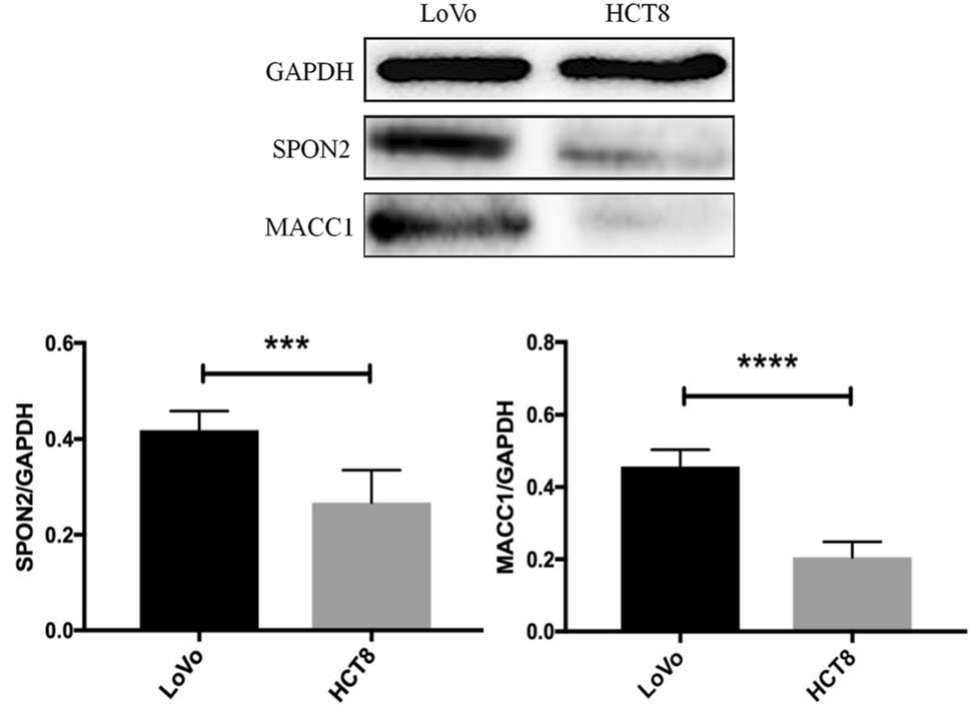
Western blot quantitative analysis of SPON2 and MACC1 in LoVo and HCT8 liver metastases, using independent sample t-test to analyze the differences between the two proteins (***P = 0.0003, ****P < 0.0001)

The protein expression levels of MACC1 (0.457 ± 0.017 and 0.206 ± 0.016) and SPON2 (0.419 ± 0.015 and 0.267 ± 0.026) in LoVo liver metastasis were higher than those in HCT8 (*P* < 0.0001, *P* = 0.0003). Our results indicate that in vivo experiments, the expression of MACC1 and SPON2 in high metastatic potential LoVo cells is higher than that of low metastatic potential HCT8 cells (Fig. 4).

### Linear regression analysis

**Fig. 5:**
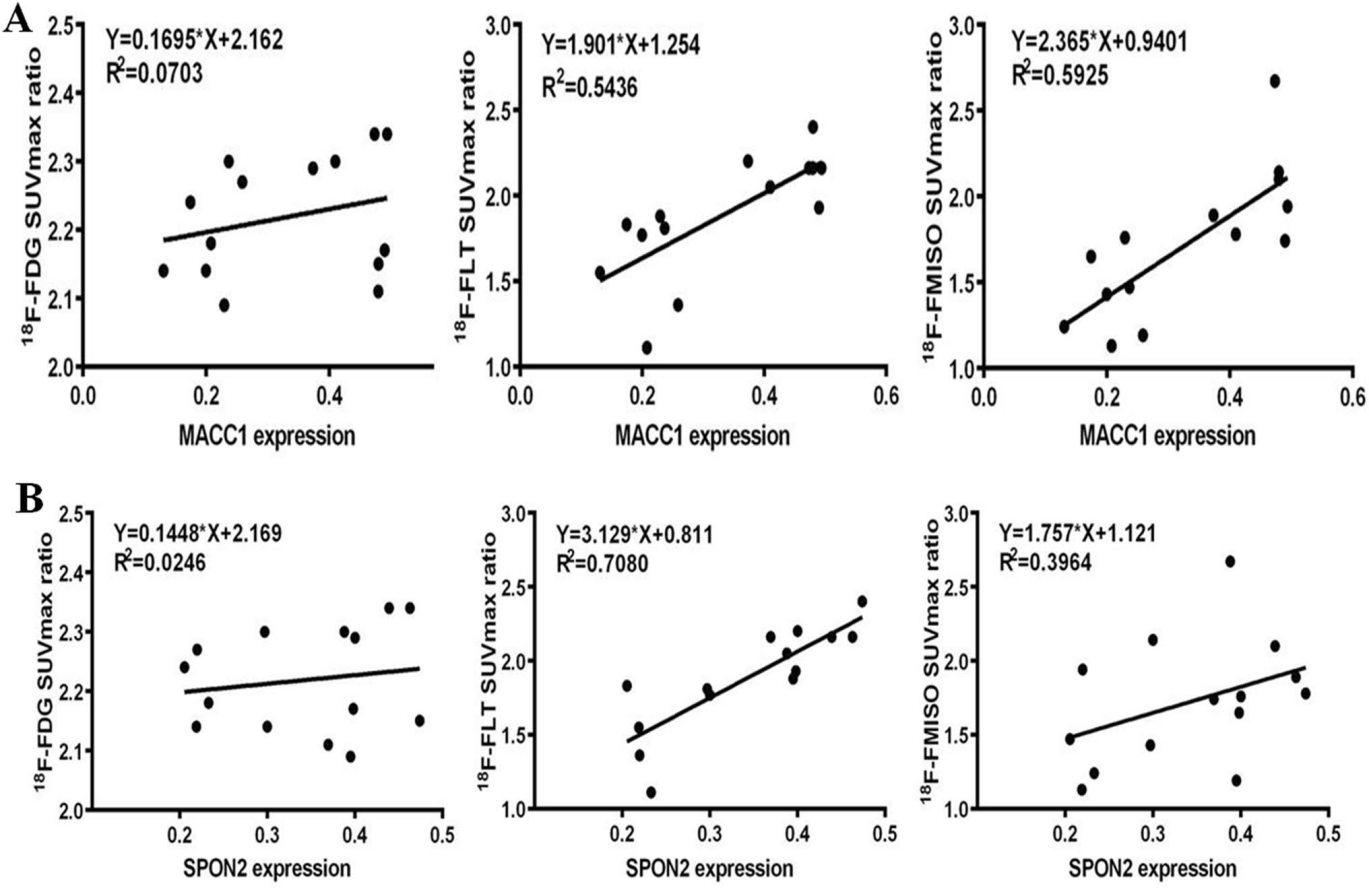
Correlation analysis between MACC1 and SPON2 and three tracers in LoVo and HCT8 liver metastases (n = 14). **a** There was a significant positive correlation between MACC1 expression and expression of ^18^F-FLT SUVmax ratio and ^18^F-FMISO SUVmax ratio in liver metastasis. **b** There was a significant positive correlation between the expression of SPON2 in liver metastases and the expression of ^18^F-FLT SUVmax ratio

**Fig. 6:**
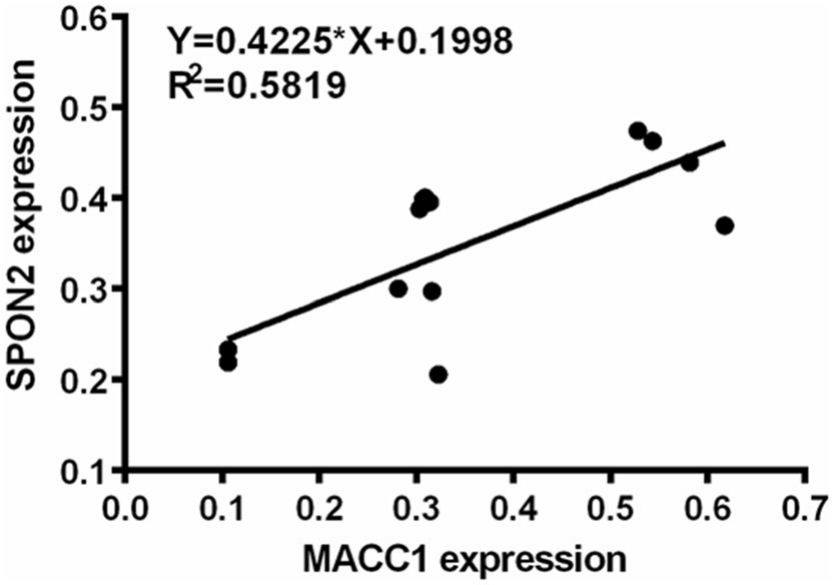
Correlation between MACC1 and SPON2 protein expression. Linear regression analysis showed a significant positive correlation between MACC1 and SPON2 (r = 0.763, *P* = 0.0015)

Figure 5 shows the correlation between MACC1 and SPON2 protein expression and PET SUVmax ratio in different cell liver metastases; Fig. 6 shows the correlation between MACC1 and SPON2 protein expression. Linear regression analysis showed a significant positive correlation between MACC1 and SPON2 (r = 0.763, *P* = 0.0015). Correlation analysis of ^18^F-FLT SUVmax ratio showed that the ^18^F-FLT SUVmax ratio was significantly positively correlated with the expression of MACC1 and SPON2 in hepatic metastasis (r = 0.737, *P* = 0.0026; r = 0.842, *P* = 0.0002). Correlation analysis of ^18^F-FMISO SUVmax ratio showed that the ^18^F-FMISO SUVmax ratio was significantly positively correlated with the expression of MACC1 in hepatic metastasis (r = 0.770, *P* = 0.0013).

## Discussion

The most central aspect of the biological behavior of colorectal cancer is invasion and metastasis, which is mediated by a series of ordered steps. Molecular imaging can visualize the metastatic process, and the living body shows changes in physiological functions at the cellular level. PET, as the main imaging device for molecular imaging, displays specific protein expression changes at the molecular level by means of specific targeting probes, which provides an effective method for visualization of tumor invasion and metastasis. In the early stage, our group established a correlation between GLUT-1 and HIF-1α by establishing molecular imaging features of liver metastasis nodules (^18^F-FMISO and ^18^F-FDG), in order to achieve early diagnosis of colorectal cancer liver metastasis [19]. However, due to the initial stage of the experiment, the selected molecular imaging indicators and tumor-related biological characteristics indicators are limited, and it is impossible to detect the biological behaviors related to tumor metastasis in many aspects. Therefore, based on this study, the new tumor metastasis markers MACC1 and SPON2 were combined to detect the physiological function of liver metastasis of colorectal cancer. The results showed that ^18^F-FLT and ^18^F-FMISO uptake finally reflected the difference in expression of tumor biomarkers (MACC1, SPON2), predicted the metastatic potential of the two tumor cells, and provided early diagnosis and metastatic potential prediction for clinical colorectal cancer liver metastasis.

In recent years, many literatures have reported on the abnormally high expression of MACC1 and SPON2 in various cancers and their potential applications in the development, progression and prognosis of cancer. In hepatocellular carcinoma, MACC1 is considered to be a biomarker of survival prognosis [21]. In lung adenocarcinoma, high levels of MACC1 expression have been shown to be associated with postoperative recurrence of tumors [23]. SPON2 has been proposed as a diagnostic biomarker for ovarian cancer and prostate cancer [23, 24]. In addition, the SPON2 gene is significantly upregulated in CRC compared to colorectal adenomas [25]. These findings of MACC1 and SPON2 effects in different malignancies support the results of this study, suggesting that they may ubiquitously promote carcinogenesis.

First, we carried out in vitro cellular uptake experiments of LoVo and HCT8 on three tracers, which laid the foundation for the detection of different biological behaviors of PET in vitro. The results showed that LoVo had higher rates of uptake of the three tracers at 240 min than HCT8, with statistically significant differences (*P* = 0.001, 0.000, 0.020, respectively). Moreover, the uptake of ^18^F-FDG is always higher than the intake of the other two tracers, regardless of cell type. PET imaging results of small animals showed that the SUVmax and SUVmax ratio values of the three tracers in LoVo cells were higher than those of HCT8, which was consistent with the results of in vitro cell uptake experiments, and further confirmed the diagnostic value of different tracer PET imaging on tumor tissues/cells. Compared with the other two tracer PET images, the FDG SUVmax and FDG SUVmax ratio values of all liver metastases were not statistically different between the two cells, which was consistent with the ^18^F-FDG in vitro uptake experiment. We speculate that this difference may be related to the internal environment, indicating that the glucose consumption of these two liver metastases is more consistent, and ^18^F-FDG cannot distinguish the difference between the two cell metastases. Most cancer cells are metabolically active, with relatively little cell proliferation and hypoxia. The net absorption of FDG is higher than FLT and FMISO in tumors. Moreover, in addition to tumor cells, many inflammatory cells are usually present in malignant lesions, resulting in higher FDG uptake [26]. The FLT SUVmax and SUVmax ratio values were both higher than FMISO, consistent with the results of the cellular uptake experiments. This suggests that FLT imaging is more sensitive to CRC liver metastasis than FMISO.

Tumor invasiveness is a key factor affecting the prognosis of colorectal cancer, including differentiation, growth rate, and metastatic potential. In this manuscript, the new biological characteristics of liver metastasis related to liver metastasis, MACC1 and SPON2, were combined to analyze the feasibility, specificity and effectiveness of multi-probe uptake on biological characteristics of metastasis from both in vivo and external aspects.

As a biological marker for the progression, metastasis and survival of many solid tumors, MACC1 has considerable basic and clinical research value [27, 28]. However, it has not been reported in the pathological mechanism of liver metastasis of colorectal cancer and clinical marker research. Western blot results showed that there was a significant difference in the expression of MACC1 between LoVo and HCT8 liver metastases (*P* < 0.0001), which was consistent with the results of immunochemical experiments. This indicates that the stronger the cell's ability to metastasize, the higher its MACC1 expression, and MACC1 can also reflect the metastatic ability of colorectal cancer cells. In this study, we found that MACC1 was significantly correlated with ^18^F-FLT SUVmax ratio and ^18^F-FMISO SUVmax ratio (r = 0.737, *P* = 0.0026; r = 0.770, *P* = 0.0013). It can be seen that MACC1, a novel tumor metastasis marker protein, can not only reflect the malignant degree of tumor, but also closely related to tumor proliferation and hypoxia metabolism. That is, the uptake characteristics of ^18^F-FLT and ^18^F-FMISO tracers can reflect the invasion and metastasis ability of colorectal cancer, and can be detected by MACC1 expression level, which has not been reported in previous studies.

SPON2 has been observed to increase SPON2 gene and protein expression in liver cancer [29], gastric cancer [30], ovarian cancer [23], and prostate cancer [18, 24, 31, 32]. Studies have shown that the SPON2 gene is upregulated in colorectal cancer compared to colorectal adenomas [25, 33]. Western blot analysis showed that there was a significant difference in the expression of SPON2 between LoVo and HCT8 liver metastases (*P* = 0.0003), which was consistent with the results of immunochemistry experiments. The stronger the cell's ability to metastasize, the higher its SPON2 expression, and SPON2 also reflects the metastatic ability of colorectal cancer cells. In this study, we found that SPON2 was significantly correlated with ^18^F-FLT SUVmax ratio (r = 0.842, *P* = 0.0002). It can be seen that the new tumor metastasis marker protein of SPON2 can not only reflect the malignant degree of tumor, but also closely related to tumor proliferation and metabolism. That is, the uptake characteristics of ^18^F-FLT can reflect the invasion and metastasis ability of colorectal cancer, and can be detected by SPON2 expression level. It can be seen that overexpression of SPON2 can enhance the proliferation of colon cancer cells. This is consistent with previous reports that SPON2 may be a biomarker for CRC diagnosis and prognosis [34].

This study shows that ^18^F-FLT and ^18^F-FMISO are more favorable for the detection of liver metastases from colorectal cancer than ^18^F-FDG. These three tracers have different metabolic pathways and reflect different physiological processes. The difference in imaging reflects differences in the levels of metabolic substrates (thymine, nitroimidazole and glucose), which may be due to microenvironment, blood. The effects of circulation, metabolism or related pathways. This study showed that the level of glucose metabolism was significantly higher than that of thymine and nitroimidazole at the cellular or tissue level, but there was no significant difference in the expression of ^18^F-FDG between cells with different metastatic potential and corresponding liver metastases. It is indicated that malignant tumors have high glucose metabolism, but glucose consumption is similar. ^18^F-FDG itself cannot distinguish the difference between different metastatic potential colorectal cancer liver metastases, which indirectly indicates that ^18^F-FDG is highly sensitive. Poor specificity. The ^18^F-FLT SUV values in both colorectal cancer liver metastasis models were higher than ^18^F-FMISO, which was consistent with the results of in vitro cell uptake experiments. It indicates that the proliferation and metabolism of tumors are higher than their hypoxia metabolism at the tissue or cell level, that is, the proliferation level can better reflect the invasion and metastasis ability of tumors. In addition, ^18^F-FMISO has lower sensitivity to ^18^F-FLT than liver metastases, but it has different degrees of correlation with tumor metastasis-related proteins. By comparing the characteristics of in vitro and in vivo nuclide uptake and the comprehensive analysis of MACC1 and SPON2 protein levels in colorectal cancer, we found that the higher the ^18^F-FLT uptake, the stronger the cell transfer ability, the higher the protein levels of MACC1 and SPON2, ^18^F-FLT The level of uptake was significantly correlated with the expression of MACC1 and SPON2. The higher the ^18^F-FMISO uptake, the stronger the cell transfer ability, and the higher the MACC1 protein level, the ^18^F-FMISO uptake level was significantly correlated with MACC1 expression. Individual differences between tumor phenotypes complicate the choice of tumor treatment options [35, 36]. As the results of this study show, ^18^F-FLT and ^18^F-FMISO PET can not only show the heterogeneity between tumors, but also have the ability to distinguish the biological characteristics of tumors. Therefore, the combination of ^18^F-FLT and ^18^F-FMISO PET imaging is also of great value for the choice of tumor treatment options.

This study has certain limitations. The experiment used ROI technology to semi-quantitatively analyze the lesions. Through visual analysis, the ROI region was drawn in the abnormal uptake area of the liver. Due to the small lesions of liver metastases in some nude mice, there was a certain error in the outline of the ROI region, and the liver metastasis model Less in quantity. In the future, it is necessary to expand the sample size and improve the experimental methods to provide greater clinical applicability for early detection, metastatic potential evaluation, and prognosis evaluation of colorectal cancer liver metastasis.

In summary, although ^18^F-FDG has higher sensitivity than the other two tracers, it lacks specificity, and the inability to distinguish the diagnosis is still a necessary disadvantage. Both ^18^F-FLT and ^18^F-FMISO imaging can evaluate the invasion and metastasis ability of colorectal cancer and can be detected by detecting the expression level of new tumor markers and multi-probe metabolic markers. It is a preferred PET tracer for the diagnosis of colorectal cancer liver metastasis.
